# Isotactic Polypropylene (iPP) Foils—Correlation of Core and Shell Crystallinity with Mechanical Properties Obtained by Nanoindentation

**DOI:** 10.3390/polym17060736

**Published:** 2025-03-11

**Authors:** Miroslav Huskić, Lidija Slemenik Perše, Boris Orel, Mohor Mihelčič

**Affiliations:** 1 Faculty of Polymer Technology, Ozare 19, 2380 Slovenj Gradec, Slovenia; miroslav.huskic@ftpo.eu; 2 TECOS, Slovenian Tool and Die Development Centre, Kidričeva 25, 3000 Celje, Slovenia; 3 Pomurje Science and Inovation Centre, Lendavska ulica 5a, 9000 Murska Sobota, Slovenia; 4 Faculty of Mechanical Engineering, University of Ljubljana, Aškerčeva ulica 6, 1000 Ljubljana, Slovenia; lidija.slemenik.perse@fs.uni-lj.si; 5 National Institute of Chemistry, Hajdrihova 19, 1000 Ljubljana, Slovenia; borisorel14@gmail.com

**Keywords:** isotactic polypropylene (iPP), foils, calendering, flash differential scanning calorimetry (Flash DSC), nanoindentation, polarized optical microscopy, crystallinity, haze

## Abstract

This study investigates the correlation between the crystallinity and mechanical properties of calendered isotactic polypropylene (iPP) foils, focusing on the influence of haul-off speed and additive type. Two groups of iPP foils produced on an industrial scale were compared: (i) foils containing 10 wt.% recycled PP at haul-off speeds of 2 and 10 m/min; and (ii) foils with different additives (neat PP, 10 wt.% recycled PP, and PP random copolymer) at a constant haul-off speed of 10 m/min. All foils exhibited a pronounced skin–core structure, with the inner surface showing higher crystallinity (up to 10%) due to slower cooling rates, as determined by Flash Differential Scanning Calorimetry (Flash DSC). Nanoindentation tests correlated these differences in crystallinity with variations in the hardness and elastic modulus across the cross-section of the foil. Higher haul-off speeds (10 m/min) resulted in increased crystallinity, a higher elastic modulus and higher hardness. Polarized optical microscopy (POM) confirmed the morphological differences and highlighted the presence of highly oriented skin layers and stratified crystalline structures. These findings emphasize the significant influence of processing conditions, such as hauling speed and the addition of recycled polypropylene or a random copolymer, on the mechanical and optical properties of iPP foils. This comprehensive approach to characterizing complex structure–property relationships is valuable for optimizing the production and performance of polypropylene-based packaging foils on an industrial scale.

## 1. Introduction

In the packaging industry, isotactic polypropylene (iPP) represents one of the most used polymers, gaining importance due to the favourable combination of price and performance and the possibility of broadening its range of properties by means of modification [1]. Many technologically relevant properties of iPP such as its optical transparency, rigidity, stability, shrinkage, hardness and elasticity, except from its composition, depend on its crystalline microstructure, which is intimately related to processing methods and manufacturing conditions [2,3].

Calendering is the most used technique in the production of foils for the packaging industry [4]. During calendering, iPP is exposed to flow and fast cooling [5], which induces a typical “skin–core” structure with various morphologies and orientations. Inevitably, the skin–core microstructure influences the mechanical and optical properties of the polymer [6,7], and this concept strongly supports the relevance of studying skin–core structures with respect to processing parameters. The surface, the intermediate layers and the core [8,9] morphology can significantly differ depending on the shape of the spherulites, their organization and the degree of crystallinity. The complexity of the iPP skin–core crystalline nature has already been demonstrated. Wang et al. [10] already reported highly oriented tread- and cylinder-like crystalline morphologies which form in iPP under various applied shear stresses and different temperatures. Recent theoretical multiscale simulation studies conducted by Ruan [11] enable the reproduction of a typical “skin–core” structure with a spherulitic structure in the core and a rod-like (or shish-kebab) structure in the skin, agreeing with experimental findings [12].

Among experimental methods used for studying the skin–core microstructure, WAXS alone and SAXS in combination with AFM [13,14] have mostly been used. The aim of our work was to use two not so often used techniques, namely flash scanning chip calorimetry (FSC) and nanoindentation, to gain an insight into the core–skin structure of iPP. The results were combined with the polarized optical microscopic (POM) measurement, which despite its simplicity provides a quite fast and efficient way for obtaining evidence about the skin–core arrangement of foils [15]. FSC enables calorimetric measurements of small amounts (from nanograms to a few μg) of the polymer at fast cooling and heating rates matching those present on industrial calendering lines [16,17,18]. Toda et al. [19] and Shawe [20] have conducted comparative studies of melting kinetics of iPP crystals obtained over a wide range of heating rates (from 10^−2^ to 10^4^ K/s) using standard and flash DSC. It has been shown that, by using FSC, the melting behaviour of polymer crystals over a wide range of heating rates could be obtained and that the reorganization of the crystals occurring at low temperatures can be avoided. Nanoindentation is a simple but effective testing method for investigating the mechanical behaviour of materials at the nanoscale [21]. In general, hardness and the elastic modulus are the most common mechanical properties determined by means of nanoindentation. For example, Jiang et al. [22] showed that nanoindentation is a very useful tool for determining the mechanical properties of the skin–core structure of micro-injection moulded iPP gears. The combination of these two techniques has recently been successfully used to determine the nanostructure of PLLA [23] and POM [24].

Understanding the relationship between the crystalline structure and the mechanical properties of iPP films is of fundamental importance for industrial applications. Therefore, many studies have been conducted to correlate and predict their properties and behaviour [25]. However, many studies have focused on characterizing the mechanical properties by determining the tensile modulus [26,27,28,29,30]. These standard tests are quite fast but can only determine the bulk properties of polymers and cannot distinguish between anisotropic structures of PP films expressing different properties in their skin–core layers.

In this study, we focused on the microscopic level to investigate the inter-relation between the morphological and mechanical properties of the surface and the skin–core structure of iPP foils. Nanoindentation measurements were performed on the surface of both sides of the foils, and the derived hardness and elastic modulus were correlated with the degree of crystallinity obtained from the flash DSC measurements of both skins. The results were correlated with the obtained degrees of crystallinity of the foils.

## 2. Materials and Methods

### 2.1. Materials

Commercial nucleated polypropylene homopolymer (PPH) (Moplen HP640J) used for extrusion and thermoforming applications of food packaging containers and bottles was purchased from LyondellBasell, Houston, TX, USA. According to the producer, the melt flow rate (MFI) (230 °C/2.16 kg) of the polymer is 3.2 g/10 min. Recycled polypropylene (R) added to PP-R-2 and PP-R-10 samples (10 wt.%) was obtained from ground self-produced iPP foils and thermoformed products. The random copolymer (RC) Tipplen R660 (added to PP-RC-10 sample) utilized as an additive for improving the optical qualities of the end product was purchased from MOL group, Budapest, Hungary.

### 2.2. Extrusion of Foils on Industrial Calendering Line

**Figure 1:**
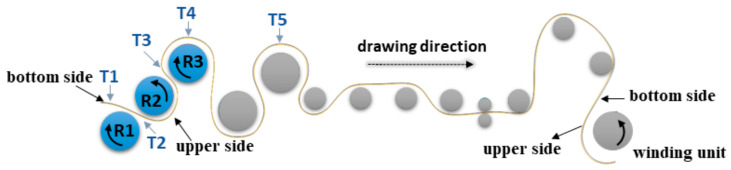
The schematic illustration of the iPP foil preparation process on a calendering line. The rollers are labelled as R_1_–R_3_ with measuring temperatures T_1_–T_5_.

All iPP foils were prepared at the company Panplast d.o.o. (Logatec, Slovenia) by using an industrial single-screw extruder (BG plast, Marnate, Italy) (L/D = 44:1, Φ = 130 mm). The process parameters and the composition of the calendered iPP foils are presented in Table 1. The production line was equipped with die of a 700 mm width and three heated calender rollers of a 500 mm diameter. The schematic illustration of the calendering line is presented in Figure 1.

Due to the high sensitivity of the iPP structure to the processing conditions, particularly the cooling rate, it is very important to monitor temperature conditions during manufacturing. The temperature of iPP polymer melting on the extrusion nozzle was 245 °C (T_1_). The temperature of the iPP foil on the calendering line was controlled by the temperature of the rollers R_1_ (T = 55 °C), R_2_ (T = 80 °C) and R_3_ (T = 64 °C) (Figure 1).

The time–temperature profile was varied by changing the rotating speed of the rollers (Table 1). The haul-off speed for sample PP-R-2 was 2 m/min, while the other three samples (PP-10, PP-R-10 and PP-RC-10) were prepared at a haul-off speed five times higher (10 m/min). The design of the experiment allowed for the investigation of two aspects: how the material structure is affected by (i) the speed of hauling (samples PP-R-2 and PP-R-10) and (ii) additives such as recycled iPP and a random copolymer (sample PP-10, PP-R-10 and PP-RC-10).

All iPP foil samples were curved in the direction of hauling. The upper side was convex and the lower side was concave. The upper side of the iPP foil is the one which is on the outer side of the foil (and calender), at the point where the foil passes between the first two rollers (R_1_ and R_2_). The curvature occurs due to the different thermal conditions to which the upper and lower sides of the foil were exposed during calendering. When the foil passed through the rollers, one side was exposed to the temperature of the roller surface which was different from that of the air.

At the point where the foil passed through the rollers, it was exposed to a pronounced temperature shock. The upper side being in contact with roller R_1_ (T = 55 °C) cooled down faster than the lower side being in contact with the warmer roller R_2_ (T = 80 °C) roller. As a result of the temperature gradient between R_1_ and R_2_, different stresses on both surfaces were frozen, which was reflected in the curvature of the foil.

As a result of the temperature gradients between the rollers (R_1_/R_2_ and R_2_/R_3_), the internal stresses generated became frozen, which we expected to be evident in the nanoindentation and crystallinity measurements. The hauling speed for the preparation of ~0.75 mm thick iPP foils is related to production capacity, which was in our case 2 m/min with a throughput of 60 kg/h of material. For samples prepared with a 5 times higher production speed, the production capacity of the foil was also 5 times higher, i.e., 300 kg/h.

In order to describe the time–temperature profile, which affects cooling gradients in addition to the time required to pass from one roller to another, we also need to know the real temperatures at selected points. Temperatures were measured as close as possible to the point where roller R_1_/R_2_ (T_2_) and R_2_/R_3_ (T_3_) meet, and at the exit of roller R_3_ (T_4_). The temperature of the foil on the winding roller was 28 °C. The temperature measurements at different points were performed using an FLIR S65 thermal imaging camera (FLIR Systems, Kings Hill, UK).

**Figure 2:**
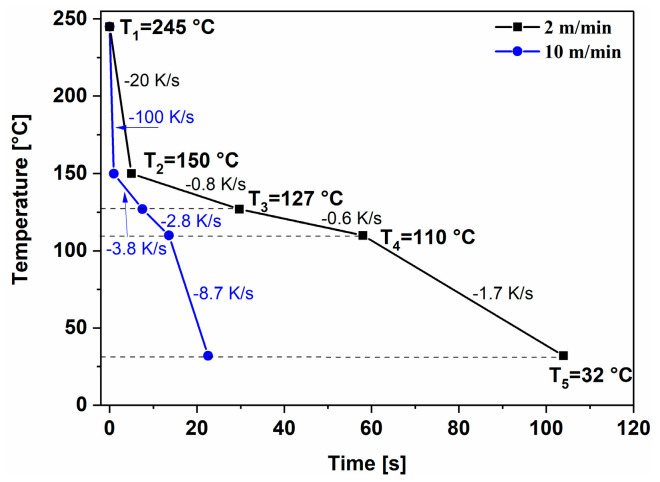
A schematic presentation of the time–temperature profile in the production line.

Examination of the two different time–temperature ramps of the prepared foils (Figure 2) showed that the cooling rates were within the measuring capacity of the FSC method used in this study.

### 2.3. Characterization Methods

#### 2.3.1. Determination of Degree of Crystallinity

The degree of crystallinity was determined by FSC. All measurements were performed on a Mettler Toledo Flash DSC 1 calorimeter (Columbus, OH, USA). Samples were cut from foil under the microscope and placed on the sensor according to the instructions reported in [16,31,32]. The samples were heated/cooled in a temperature range of 15–195 °C with a heating/cooling rate of 1000 K/min, except for mass determination, where the cooling rate was 30 K/min. The holding time at both temperatures was 0.1 s. The unknown mass of the samples measured by flash DSC (*m*_FSC_) was obtained from the measured enthalpies (∆*H*) of the melting peaks obtained by DSC (*H*_DSC_) and flash DSC (*H*_FSC_) according to Equation (1):

(1) $$m_{FSC}={∆H}_{FSC}\times (\frac{m_{DSC}}{∆H_{DSC}})$$

The typical sample weight was 100-150 nanograms. The degree of crystallinity was calculated by using the melting enthalpy of 100% crystallized ($∆H_{m}^{0}$) PP, namely 207 J/g [33].

The crystallinity of the foil surface on the side that comes into contact with the roller with a higher temperature (R2, T = 80 °C) (Figure 1) (inner surface) and the foil surface on the opposite side (R1, T = 55 °C) (upper surface) was measured. DSC measurements were performed on a Mettler Toledo DSC 2 calorimeter (Columbus, OH, USA) in a temperature range of 15–195 °C. The sample mass was 5–10 milligrams. Nitrogen flow was 20 mL/min and the heating/cooling rate was 30 K/min.

#### 2.3.2. Polarized Optical Microscopy (POM)

The morphology of the cross-section of the extruded films was examined using polarized transmitted light on a Leica DM4000M (Keyence, Osaka, Japan) microscope. The specimens were prepared by using a thin-layer polishing method. The specimens were encapsulated in a special resin and sanded with sandpaper with a grit size up to 2400. They were then bonded to a cover glass with the same resin, cut and ground again to form a thin layer. Two different POM measurements were performed: one with the foil’s extrusion direction oriented along the polarization direction of impinging light (0°) and the second one, with an extrusion direction, inclined at 45 degrees to the direction of polarized light (45°). The lightness and contrast of the measured POM micrographs were adjusted to obtain the clearest view of different cross-section layers and their stratification.

#### 2.3.3. Nanoindentation

All nanoindentation measurements were performed by using a Nanoindenter G200 XP instrument manufactured by Agilent Technologies (Santa Clara, CA, USA), equipped with a standard three-sided pyramidal Berkovich probe. Measurements were performed at room temperature using the CSM (Continuous Stiffness Measurement) method with a tip oscillation frequency of 45 Hz and a 2 nm harmonic amplitude [34,35].

**Figure 3:**
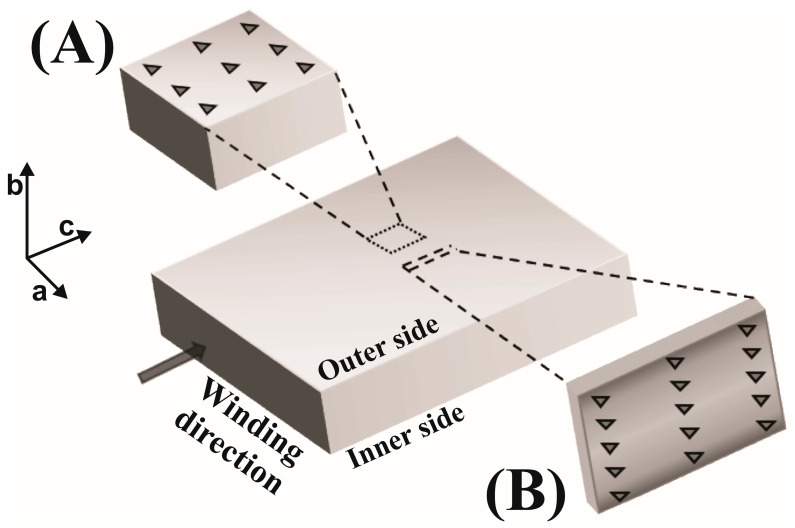
A schematic representation of the nanoindentation measurements performed on two different foil planes: (**A**) plane ac normal to the foil surface (top left) and (**B**) the cross-sectional plane bc that was cut out of the foil. The symbol (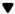) indicates the indents of the measurements.

Two different sets of nanoindentation measurements were performed (Figure 3). The first—measurements A—were made on the inner and outer (Figure 1) surfaces of the foils (ac plane). Additionally, measurements B were taken on the (bc) cross-section of the foils with the aim of obtaining values of the elastic modulus and hardness at different points of the corresponding cross-section. The values of the elastic modulus and hardness obtained from measurements A on the ac plane were then correlated with the degrees of crystallinity of the inner and outer parts of foils obtained from the FSC measurements. The results thus obtained enabled the construction of the calibration curve which was then used for obtaining the degrees of crystallinity for the sites on the (bc) cross-section of the foils, which were not directly measured by FSC. The information about the degrees of crystallinity enabled us to correlate, at least qualitatively, the crystallinity of the foils obtained from the POM measurements.

To characterize the foil surface during measurements A, 36 tests were performed for each foil with a 100 µm distance between adjacent indentations to exclude interaction effects (Figure 3A). The measuring depth was 2000 nm and the average value in the range of 800 to 2000 nm depth was used for data analysis. No surface treatment like polishing was performed on the inner and outer surfaces of foils.

Cross-section nanoindentation (measurements B) was performed in the “scanning” regime, which requires a special sample preparation procedure. Calendered foils were cut into small pieces in the extrusion direction and afterwards embedded in epoxy resin. The surface of each sample was smoothened using sandpaper P4000 and polishing suspension prior to characterisation. Measurements were performed using the same CSM method as for measurements A. The indentation depth was 4000 nm with a distance of 100 µm between each indent in the horizontal direction and 91–107 µm in the vertical direction depending on the thickness of the foil. Locations of the indents were positioned to form a mesh of 12 × 6 data points (Figure 3B). The analysis was performed for the range of depth from 3000 to 3500 nm in order to avoid surface effects caused by polishing the samples. The obtained values were interpolated into the mappings of the modulus and hardness using the coordinates of the film cross-section with the use of MATLAB R2018b software [36].

#### 2.3.4. Optical Properties

The optical properties—haziness (H), total (TT) and diffuse total (DT) transmittance—of calendered iPP foils were determined using a Lambda 950 UV-Vis spectrometer (Perkin Elmer, Hopkinton, MA, USA) equipped with an integrational sphere. Measurements were performed according to the ASTM test method D1003 [37]. According to the definition, the haze (%) is a ratio of the diffuse total transmittance (DT (λ)) to the total transmittance (TT (λ)) in the range between 380 and 780 nm.

## 3. Results and Discussion

### 3.1. DSC Measurements

DSC measurements, performed at a heating/cooling rate of 30 K/min, were performed for sample mass determination with flash DSC. In order to construct a calibration curve for a wide range of crystallinity values, an additional sample with a higher degree of crystallinity was prepared by annealing (PP_ann_). This sample was prepared by melting iPP at 245 °C, which was then cooled to 100 °C, annealed for 3 h at this temperature and then cooled to room temperature at a cooling rate of 0.1 K/min.

In the first step, we correlated the DSC results (Table 2) obtained from the first heating phase at 30 °C/min for samples PP-R-10 and PP-R-2, which had the same composition and were prepared at different drawing speeds (haul-off speed PP-R-10 > PP-R-2, Table 1). The corresponding Χ_c_ value of PP-R-10 (41.7%), prepared at a higher drawing speed, was higher than the Χ_c_ value of the slower drawn PP-R-2 (39.3%) foil.

In the second step, we compared the DSC results of iPP foil samples PP-10, PP-R-10 and PP-RC-10 which were processed at the same speed but with different compositions. The results from the first heating phase show that the PP-10 foil without additives (Table 2) was the most crystalline foil (43.4%). The addition of the additives lowered the crystallinity of PP-R-10 and PP-RC-10 foils by 1.7% and 3.9%, respectively. Sample PP-RC-10 exhibited the lowest Χc value, as the addition of a random copolymer (RC) hindered the formation of a well-ordered crystalline phase, thereby improving the clarity and optical properties of the polymer foil. As expected, the highest Χ_c_ values were obtained for the annealed PP_ann_ sample, where annealing promoted crystallization by providing sufficient time for chain mobility, reducing the amorphous content, thickening the lamellae and thus increasing crystallinity.

During the second heating phase, all foils showed higher Χ_c_ values compared to those obtained during the first heating phase, and their relative values reflected differences in their composition, but not in their specific morphology, which were erased during the first heating phase. As expected, the highest degree of crystallinity was obtained for PP-10 and the lowest for PP-R-2. The latter can be explained by the longer retention time of iPP in the extruder at a high temperature, which causes the decomposition of the polymer [38].

### 3.2. Flash DSC Measurements

To gain further knowledge about the crystallization behaviour of calendered iPP foils, the main focus was to determine the degree of crystallinity (X_c_) of both skins (Table 3). We expected that due to the multi-roller system, the crystallization conditions encountered for both sides of the foil were not exactly the same since the first two rollers had different temperatures (55 °C vs. 80 °C). This asymmetry in temperature likely led to different X_c_ values of the inner and outer skin.

The cooling of the foils on the industrial line (Figure 1) and thus the conditions under which crystallization took place were not the same for the outer and inner sides of the foils. The foil coming out from the extruder first touched the outer surface of roller R_1_, where the temperature is lower (55 °C) than the temperature of roller R_2_ (80 °C). When the foil was in contact with roller R_2_, its outer surface was exposed to the outer environment (air, ambient temperature), which has a lower temperature than the inner side of the foil (80 °C). However, it is not only the temperature difference that is important, but also the thermal conductivity, which is much better in steel than in air. Here, we are facing significant asymmetry of the temperatures at the outer and inner sides of the foil, thus creating differences in the conditions under which the foils are cooled and crystallized. Due to the more intense cooling of the outer side of the foil, its degree of crystallinity ($X_{c}^{outer}$) was lower than the degree of crystallinity of the inner side ($X_{c}^{inner}$).

Since the foils PP-R-2 and PP-R-10 were nominally identical in their composition (addition of ~10 wt.% of recycled polypropylene) but were drawn at different speeds (2 m/min vs. 10 m/min), an explanation for the higher crystallinity of PP-R-10 can be given by the higher shear-induced nucleation as a result of the higher drawing speed. However, since the results of the second heating phase, measured by DSC, are also higher, we cannot exclude the small thermal degradation of the polymer in the extruder due to the longer retention time on the heated rollers. The results also agree with the findings of Fisher and Drummond [39], who found that the crystallinity is lower in foils that are pulled slower over cylinders at temperatures similar to ours (55–80 °C).

The interpretation, which includes only differences in iPP compositions but does not take into account the shear forces that occur during calendering, applies to the foils PP-10, PP-R-10 and PP-RC-10. As expected, the PP-10 foil without the addition of additives exhibited the highest difference between the degree of crystallinity of the inner and outer part of the foil. Slightly lower values were observed for the foil PP-R-10, which contained a recyclate of the same material. The results for the foil PP-RC-10, which contained a random copolymer, were much lower. This can be explained by the possibility of recrystallization during DSC measurements which cannot occur during FSC measurements.

### 3.3. Nanoindentation Measurements: Dependence of Mechanical Properties of Outer and Inner Sides of Foils

In addition to the chemical composition, the hardness and modulus of elasticity of iPP films depend on the crystallinity (X_c_) of the polymer, and the latter on the method of processing and the processing conditions [2,3]. It has been shown [40,41] that nanoindentation measurements are selective enough to determine changes in the local values of the modulus of elasticity and hardness on the surface of objects. This was clearly demonstrated by nanoindentation studies performed for objects manufactured by micro-injection moulding (MIM) [42]. In the case of MIM, the effect of shear forces and temperature on the surface morphology is large, which leads to significant differences in the mechanical properties of the inner part of the polymer and its surface. In this study, we therefore wanted to show if there are differences in the mechanical properties of the outer and inner surfaces of the foils which were extruded under milder conditions (Table 3).

The results of the FSC measurements (Table 3) reveal that molten iPP exposed to various processing conditions on the calendering line (Figure 1) exhibited locally different degrees of crystallinity (X_c_) for the inner and outer surfaces. If the mechanical properties depend on the crystallinity even at the local level, then there should be a simple relationship between the measured values of X_c_ and the corresponding values of the modulus of elasticity and hardness for all points measured by nanoindentation.

**Figure 4:**
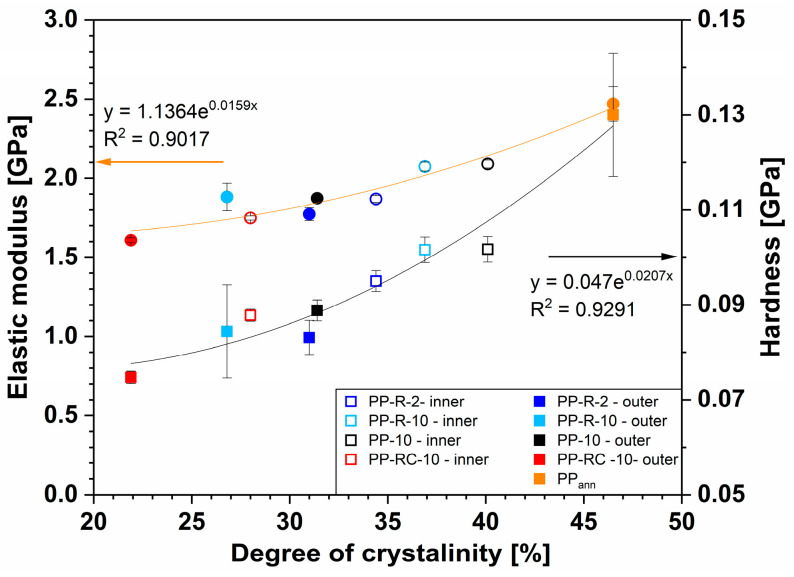
The modulus of elasticity and hardness obtained from the nanoindentation measurements at different locations on the surface (inner and outer) of the iPP foils in relation to the crystallinity values determined from the FSC measurements.

The results of the surface mechanical properties independent of the degree of crystallinity (Table 3), regardless of the composition (Figure 4), reveal a dependence of local mechanical properties (elastic modulus and hardness) on the crystallinity values obtained from the FSC measurements. The annealed PP (PP_ann_) sample showed the highest values of crystallinity (46.5%), modulus (2.47 ± 0.05 GPa) and hardness (0.13 ± 0.005 GPa). The correlation appears to be exponential, although the linear fit to the data is not much worse (R^2^ = 0.88 and 0.92 for modulus and hardness, respectively). The modulus showed a more significant increase with increasing crystallinity (53% from the lowest to the highest value) compared to the hardness values (28%). A good match confirmed that the differences obtained were attributed to the different calendering speeds and temperature conditions on the industrial processing line. However, it is worth noting that both linear and exponential dependence [43] of mechanical properties could be found in the literature. The differences between linear and exponential fittings, determined for our samples, were not significant; however, the exponential fit more accurately described the wider range of the obtained results. Nevertheless, Mandelkern and Popli showed that the linear fit is valid but not across the whole range of crystallinities [44].

**Figure 5:**
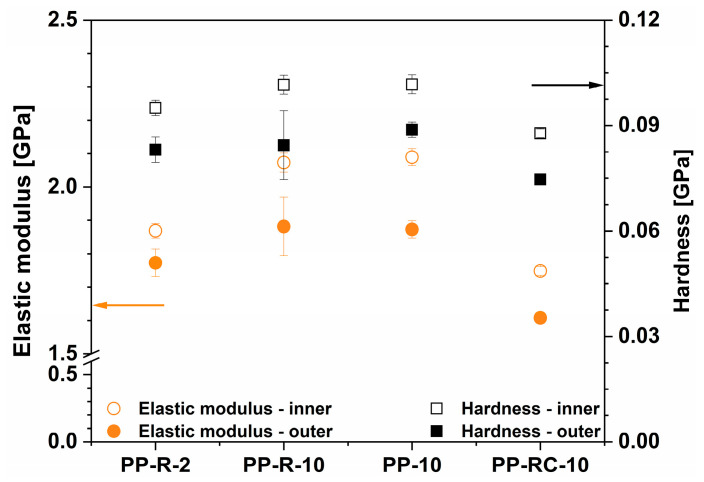
Elastic modulus and hardness obtained from nanoindentation measurements on inner and outer side of foils.

The results presented in Figure 4 show a good correlation of surface mechanical properties with crystallinity, regardless of the composition, the processing parameters and the side of the foil. Some scattering of the results (lower R^2^) can be explained by the influence of different residual stresses. The melts differed in structure and therefore in viscosity. In addition, the cooling rate was different at both sides of the foils. The residual stresses that remained frozen in the foils were different from sample to sample and between the inner and outer side. It is known that the residual stresses influence the indentation measurements and that indentation can also be used to determine them [45]. The influence of processing parameters on the crystallinity, nanoindentation modulus and hardness is shown in Figure 5.

### 3.4. Morphology Studies: POM and Nanoindentation Measurements

In order to gain a better understanding of morphological properties in relation to processing conditions, polarized optical microscopy (POM) measurements of PP-R-2 and PP-R-10 foils were performed, and the corresponding micrographs were compared to the distribution of the local mechanical properties obtained from nanoindentation measurements.

#### 3.4.1. POM and Nanoindentation Measurements of Cross-Sections

In order to obtain information about the effect of processing conditions (temperature and shear) on the morphology (i.e., preferred crystallite shape, orientation and size) of foils, POM measurements were performed. In the absence of quiescent conditions, the polymer crystallizes homogeneously and there are no differences between the surface and the core [46]. It has been shown many times [8,9,47,48,49] and theoretically proved with the Janeschitz–Kriegl model that under flow conditions, molecules elongate in the direction of flow and form threads or a shish form [50]. As the flow stops, kebabs consisting of lamellae grow radially from the shish form. The result is a layer called the highly oriented skin.

**Figure 6:**
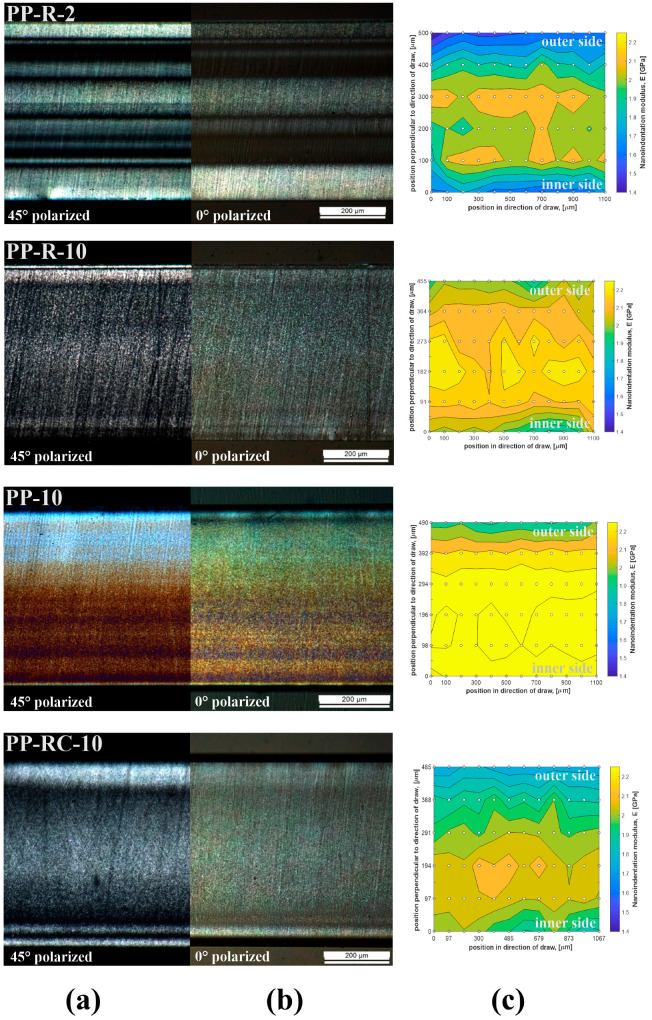
POM micrographs and nanoindentation measurements of hardness modulus distribution for PP-R-2, PP-R-10, PP-10 and PP-RC-10 foils. Comparison of POM micrographs in 45° (**a**) and 0° (**b**) polarization direction and (**c**) distribution of hardness modulus at different sites on same cross-section as used for POM micrographs.

In general, when observed in transmitted polarized light, the polymer displays bright (Figure 6a; 45° polarization) or dark (Figure 6b; 0° polarization) areas depending on the angle between the oriented direction and the polarizer and analyser [51]. Studies of polymeric objects prepared by micro-injection moulding (MIM) provided clear evidence of the correlation between object morphology and processing conditions (temperature and shear). Processing conditions strongly affect the oriented skin layer thickness, which depends on the polymer melt temperature and varies inversely with temperature. It has already been revealed [52] that at lower levels of shear stress, types of oriented structures other than oriented skin form, known as sausages and skin lines. They are seen as lines of relatively high orientation, and in between them, non-oriented regions exist which can be significantly wide in the case of sausages. The fact important for this study is that grained layers consisting of smaller spherulites can be observed at less intense flow rates in the middle part of the foil, as was also reported by Woodward et al. [53].

**Figure 7:**
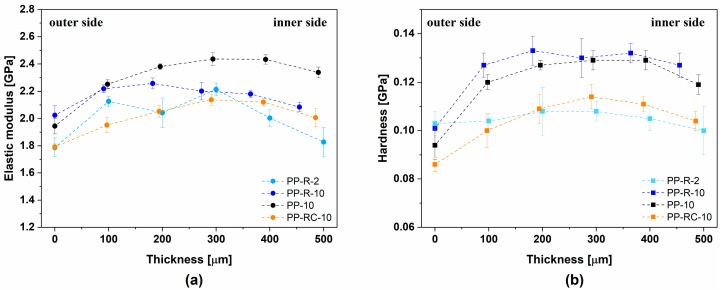
The average elastic modulus (**a**) and hardness (**b**) obtained from the nanoindentation cross-section measurements of the samples.

In the following, POM micrographs of the iPP foils, which were examined in 0° and 45° polarization directions, and oriented skin and other spherulitic structures are presented. In parallel, and supported by the results disclosed in Figure 6a,b, nanoindentation measurements were performed on the same cross-sections of the iPP foils, providing the distribution of the mechanical modulus for specific locations on selected cross-sections (Figure 6c) of iPP foils. The average values of the elastic modulus and hardness obtained from the nanoindentation measurements are summarized in Figure 7.

#### 3.4.2. Comparison of POM and Nanoindentation Measurements of PP-R-2 and PP-R-10 Foils (Different Processing Conditions)

Foils PP-R-2 and PP-R-10 were examined first, as they had the same nominal composition but were drawn at different speeds during processing. Both foils showed highly oriented skins on both sides, followed by darker, alternating areas which lead towards the crystalline core (Figure 6). However, sample PP-R-2 had a significantly more pronounced stratified layered structure with sharp transitions between different crystalline regions. Importantly, the distribution of different stratified layers in the outer and inner part of the foil was not symmetric, indicating that the crystallization conditions were not the same for the outer and inner part of the foil, agreeing with the *X_c_* values obtained from the FSC measurements (Table 3).

Although the POM measurements were not quantitative in nature, the features seen in the POM micrographs could be to some extent correlated with the *X_c_* values obtained from the FSC measurements. For example, for the PP-R-10 foil, the *X_c_* values (*X_c_*^outer^ (26.8%) and *X_c_*^inner^ (36.9%)) signalled that the crystallinity of the core was higher than that of the skin, which is not distinctly visible from Figure 6a,b.

The most stratified and distinct layers were observed for the PP-R-2 foil, contrasting the observed skin–core morphology of the PP-R-10 foil. Such stratification was expected because, as mentioned by Zhang et al. [49], the orientation degree gradually decreases as the distance from the surface increases but even the core can exhibit a certain degree of orientation, as we observed for all our samples as well. The highest orientation is reserved for the skin layers and the layers next to them.

It is clear that the asymmetrical stratification of layers could be correlated with the non-symmetrical temperature gradients on the production line causing the crystallization of the outer part to become different with respect to the inner part of the foil. Nonetheless, the different *X_c_* values of the outer and inner part of the foil surface (*X_c_*^inner^ > *X_c_*^outer^) (Table 3) confirm that the inner part of the PP-R-2 foil was for a longer time in contact with the warmer R_1_/R_2_ roller (80 °C) compared to the outer part, which was exposed to ambient air temperature. The massive crystalline layer observed at the inner part of the foil independently supports the *X_c_* values obtained from the FSC measurements, i.e., *X_c_*^inner^ (34.4%) > *X_c_*^outer^ (31.0%).

Sample PP-R-2 was hauled 5 times slower compared to all other samples, which means that (i) it was subjected to smaller shear loading with a slower rate; (ii) its temperature history is different compared to other samples. Particularly important here is the cooling rate, which was lower at every stage of travelling through the rollers, as visible from the decline in the temperature profiles in Figure 2. This means that the crystalline structure in the sample core had more time to form larger crystals, which are visible in Figure 6 as bright regions. Nevertheless, the speed of the foil was high enough to result in a change in temperature and cooling rate (alternating contact with the roller and air), and therefore, the darker regions are also clearly visible. It is important to emphasize that the highly oriented skin was present in both samples, though it was thinner for PP-R-2. Obviously, the temperature also drops because stress-induced crystallization of the skin had enough impact on the formation of the highly oriented structure even at lower hauling speeds but resulted in the formation of a thinner oriented layer compared to faster hauled samples.

Nanoindentation measurements illustrate the cross-sectional distribution of the elastic modulus measured at different sites on the cross-section areas (Figure 6; PP-R-2-C, PP-R-10-C) of foils PP-R-2 and PP-R-10. Inspection of the distributions of local mechanical moduli reveals the differences on the outer and inner part of the foils. On the outer side, the modulus was the smallest and increased towards the middle of the foil where it reached its maximum, from which its value decreased again towards the inner surface. The outer part of the PP-R-2 foil shows smaller values of the elastic modulus compared to the values on the inner side, which agrees with the fact that the crystallization conditions at the warmer part in contact with the R_2_ roller are more favourable for crystallization. Nonetheless, this could also be conceived from the *X_c_* values (Table 3). For the PP-R-10 foil, the distinction between the values of the modulus on the inner and outer side was less apparent. For this foil, the region of the highest elastic modulus values was shifted towards the (lower) inner surface as expected and observed from the POM micrographs, but for the PP-R-2 foil, the region of the maximal values of the elastic modulus was not so clear-cut.

Nonetheless, the most convincing evidence for the reliability of the results obtained from the nanoindentation measurements was obtained by comparing *X_c_* values at the outer and the inner part of the foil; the region of the higher values of the elastic modulus extended closer to the outer surface when compared to the distribution of values at the lower (i.e., inner) part of the foil. This matches well with the measured values of the degree of crystallinity for the PP-R-2 foil. Reversed *X_c_* values determined from the FSC measurements fit well with the POM micrographs and the values of elastic modulus obtained from the nanoindentation measurements.

#### 3.4.3. Comparison of POM and Nanoindentation Measurements of PP-10, PP-R-10 and PP-RC-10 Foils (Effect of Additives)

The PP-10 foil was prepared with no additives and corresponds to the original iPP polymer. A characteristic feature of this foil is the well-expressed difference in morphology as determined from the POM measurements (Figure 6, PP-10-A,B). A variation in the degree of crystallinity was observed for the outer and inner skin and core, i.e., *X_c_*^outer^ (31.4%) and *X_c_*^inner^ (40.1%).

Even though the sample PP-10 can be considered the most homogeneous in respect to both crystalline structure and nanoindentation modulus, even here highly oriented skins were observed. The layered structure was present as well (however, less pronounced) and shish–kebab layers were seen on both sides of the foil.

Both samples PP-R-10 and PP-RC-10 have additives composed of smaller molecules compared to the pure iPP (PP-10). In the case of PP-R-10, it comprises recycled iPP, which according to the literature [54], after several recycling phases, reduces in molecular length due to chain scission. As for PP-RC-10, the random copolymer is also a PP with a structure that should nucleate the crystallization process and improve the optical properties of the material. In Figure 6, we see that PP-R-10 and PP-RC-10 have a similar asymmetric structure consisting of the same elements: highly oriented skins, darker transition regions, a highly crystalline core and more layered parts at the bottom, which again ends with an oriented skin. It is true that the intensity of the POM images is different and the values of the elastic modulus obtained for PP-R-10 are higher in the core. This is due to the differences in the additives which generally lead to the formation of a topologically similar foil structure.

The distribution of the nanoindentation modulus values throughout the cross-section of PP-10 is the most homogeneous compared to all other samples. Similar to the other samples, a softer layer on the outer surface was observed, and the values of modulus increased towards the core and stabilized closer to the lower skin. On the bottom side, a softer layer was not observed. However, the asymmetric distribution of the values of the elastic modulus of foil PP-10 agrees with the fact that the crystallization condition was more favourable close to the warmer R_2_ roller surface, as was also found for the other foils. In this respect, the PP-10 foil did not behave like the other foils.

Nanoindentation measurements of foil PP-RC-10 demonstrated a similar distribution of the nanoindentation modulus compared to PP-R-10. This distribution matches well with the change in the measured *X_c_* values (Table 3), where we found that *X_c_*^inner^ (28.0%) > (*X_c_*^outer^ (21.9%). The position of the highest degree of crystallinity is shifted towards the inner surface of the foil due to the more favourable crystallization conditions in the lower inner part of the foil.

To sum up, the morphology of all foils determined from the POM measurements was in good correlation with the degrees of crystallinity obtained from the FSC measurements and with the hardness modulus measurements obtained from the nanoindentation measurements.

### 3.5. Optical Measurements of iPP Foils

**Figure 8:**
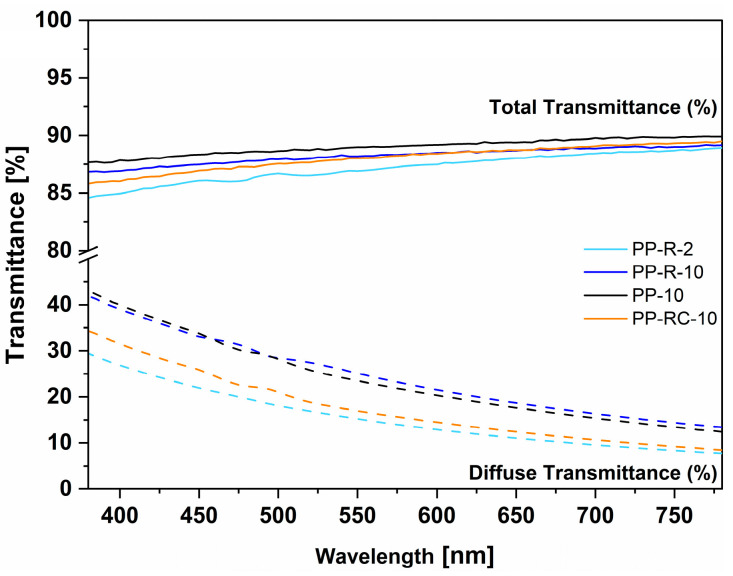
Total and diffuse transmittance (in %) spectra of iPP foils.

The difference between the total transmittance of all foils studied was small (lower than 2.2%, Figure 8, Table 4). The highest transparency was observed for the PP-10 foil and PP-R-10 (89.0% and 88.3%, respectively). This could be the result of their structure, which was quite homogeneous and compact without observable voids and stratified layers with different crystallinities. Both foils also exhibited the highest crystallinity, which did not inhibit the high total transmittance (TT) of the foils. As expected, the PP-10 and PP-R-10 foils exhibited the highest haze, agreeing with their degrees of crystallinity which were the highest among all foils.

On the contrary, the PP-R-2 foil had the lowest total transmittance (87.2%) but its corresponding haze and diffuse transmittance were only 15.8% and 14.3%, respectively. This could be correlated with the lower degrees of crystallinity of PP-R-2 and the presence of stratified crystalline layers, as observed from the POM measurements (Figure 6). For the PP-RC-10 foil, the haze was also small (17.6%) and could be explained by the presence of the nucleating ability of the clarifying agent leading to the formation of smaller crystallites with a smaller size. We plan to conduct further research in the future to obtain a clearer view of the optical properties of the examined foils.

## 4. Conclusions

The structure of the iPP foils prepared under various processing conditions (draw ratio) and with slightly different compositions (addition of recyclate or copolymer) was determined by FSC, nanoindentation and POM measurements. The morphology of the foils and their crystalline structure were determined by POM measurements. A good correlation between the layers observed in 0° and 45° polarization directions was obtained, revealing a layer-like morphology with an expressed oriented skin, shish–kebab structures and lines. The most interesting finding was the stratified layered structure of the PP-R-2 foil differing with respect to the skin–core structure of the PP-R-10 foil. The fact that these foils had the same nominal composition enabled us to ascribe the differences in the observed morphology of the PP-R-2 and PP-R-10 foils to the different cooling speeds and shear stresses.

The fact that foils with different compositions did not exhibit the same morphology was expected. At first, all foils were directly obtained from the production line and some deviations in their properties should be anticipated. However, even though the same type of iPP (MOPLEN) was used for the production of the foils, the morphology depended on the effective (average) molecular weight of the polymer, which varied in the foils studied due to the presence of additives (recyclate or random copolymer); the modified foils exhibited different structures compared to the virgin polymer. This resulted in altered viscoelastic properties and different behaviours of the polymers during stretching and cooling. Consequently, the orientation of the molecules and the residual stresses were different.

The morphology also varied across the cross-section of the foils and confirmed the existence of a skin–core morphology. The results also show that the oriented skin layer was present in all samples studied, but its thickness seemed to depend on the chemical composition of the foils and the temperature of the drawing rollers. In all foils, the central part (core) had a higher elastic modulus and hardness compared to the oriented skin. Moreover, the distribution of different layers in the outer and inner parts of the foil was not symmetrical, indicating that the crystallization conditions in the outer and inner parts of the foils were not the same. This is in agreement with the values of the elastic modulus and hardness obtained by nanoindentation measurements.

## Figures and Tables

**Table 1:** Process parameters of iPP foils produced on industrial calendering line at Panplast Company. Materials: PP-10: PPH—virgin PP homopolymer (Moplen) without additives; PP-R-2 and PP-R-10: R—recycled PP; PP-RC-10: RC—random copolymer (Tipplen).

Sample	Haul-Off Speed [m/min]	Material	Average Thickness (μm)
PP-R-2	2	PPH + R	780 ± 10
PP-R-10	10	PPH + R	720 ± 20
PP-10	10	PPH	750 ± 15
PP-RC-10	10	PPH + RC	785 ± 10

**Table 2:** Enthalpies of melting (∆H_m_), peak temperatures of endothermic melting curves (T_m_) and degrees of crystallinity (Χ_c_) for all iPP foils obtained by DSC with heating rate of 30 °C/min.

Sample	1st Heating			2nd Heating		
	∆*H_m_* [J/g]	*T_m_* [°C]	*X_c_* [%]	∆*H_m_* [J/g]	*T_m_* [°C]	*X_c_* [%]
PP-R-2	81.3	164.5	39.3	90.4	162.3	43.7
PP-R-10	86.3	170.9	41.7	93.6	165.6	45.2
PP-10	89.8	169.5	43.4	97.8	166.8	47.6
PP-RC-10	81.8	170.4	39.5	90.1	164.7	43.5
PP_ann_	100.4	167.2	48.5	93.4	161.7	45.1

**Table 3:** The melting enthalpy, melting temperature and degree of crystallinity measured at the inner and outer side of the foil determined by FSC.

Sample		Inner Side	Outer Side	∆_cryst._ [Inner − Outer]
PP-R-2	∆*H_m_* [J/g]	71.3	64.2	
	*T_m_* [°C]	147.2	141.0	
	*X_c_* [%]	34.4	31.0	3.4
PP-R-10	∆*H_m_* [J/g]	76.4	55.5	
	*T_m_* [°C]	147.5	142.8	
	*X_c_* [%]	36.9	26.8	10.1
PP-10	∆*H_m_* [J/g]	83.0	65.0	
	*T_m_* [°C]	147.0	140.5	
	*X_c_* [%]	40.1	31.4	8.7
PP-RC-10	∆*H_m_* [J/g]	58.0	45.3	
	*T_m_* [°C]	147.1	148.4	
	*X_c_* [%]	28.0	21.9	8.7

**Table 4:** Total and diffuse transmittance and haze values of iPP foil samples.

Sample	Total Transmittance [%]	Diffuse Transmittance [%]	Haze [%]
PP-R-2	87.2	14.3	15.8
PP-R-10	88.3	23.6	26.1
PP-10	89.0	22.3	24.4
PP-RC-10	88.2	16.0	17.6

## Data Availability

Data are contained within the article.
